# Acute induction of anomalous and amyloidogenic blood clotting by molecular amplification of highly substoichiometric levels of bacterial lipopolysaccharide

**DOI:** 10.1098/rsif.2016.0539

**Published:** 2016-09

**Authors:** Etheresia Pretorius, Sthembile Mbotwe, Janette Bester, Christopher J. Robinson, Douglas B. Kell

**Affiliations:** 1 Department of Physiology, Faculty of Health Sciences, University of Pretoria, Arcadia 0007, South Africa; 2 Faculty of Life Sciences, The University of Manchester, 131, Princess Street, Manchester M1 7DN, Lancs, UK; 3 School of Chemistry, The University of Manchester, 131, Princess Street, Manchester M1 7DN, Lancs, UK; 4 The Manchester Institute of Biotechnology, The University of Manchester, 131, Princess Street, Manchester M1 7DN, Lancs, UK; 5 Centre for Synthetic Biology of Fine and Speciality Chemicals, The University of Manchester, 131, Princess Street, Manchester M1 7DN, Lancs, UK

**Keywords:** bacterial lipopolysaccharide, plasma, thromboelastography, electron microscopy, amyloid, fibrin

## Abstract

It is well known that a variety of inflammatory diseases are accompanied by hypercoagulability, and a number of more-or-less longer-term signalling pathways have been shown to be involved. In recent work, we have suggested a direct and primary role for bacterial lipopolysaccharide (LPS) in this hypercoagulability, but it seems never to have been tested directly. Here, we show that the addition of tiny concentrations (0.2 ng l^−1^) of bacterial LPS to both whole blood and platelet-poor plasma of normal, healthy donors leads to marked changes in the nature of the fibrin fibres so formed, as observed by ultrastructural and fluorescence microscopy (the latter implying that the fibrin is actually in an amyloid β-sheet-rich form that on stoichiometric grounds must occur autocatalytically). They resemble those seen in a number of inflammatory (and also amyloid) diseases, consistent with an involvement of LPS in their aetiology. These changes are mirrored by changes in their viscoelastic properties as measured by thromboelastography. As the terminal stages of coagulation involve the polymerization of fibrinogen into fibrin fibres, we tested whether LPS would bind to fibrinogen directly. We demonstrated this using isothermal calorimetry. Finally, we show that these changes in fibre structure are mirrored when the experiment is done simply with purified fibrinogen and thrombin (±0.2 ng l^−1^ LPS). This ratio of concentrations of LPS : fibrinogen *in vivo* represents a molecular amplification by the LPS of more than 10^8^-fold, a number that is probably unparalleled in biology. The observation of a direct effect of such highly substoichiometric amounts of LPS on both fibrinogen and coagulation can account for the role of very small numbers of dormant bacteria in disease progression in a great many inflammatory conditions, and opens up this process to further mechanistic analysis and possible treatment.

## 1. Introduction

‘LPS’ describes a variety of cell wall lipopolysaccharides shed by Gram-negative bacteria; also known as ‘endotoxin’, they have been found in various fluids, including whole blood (WB). The ‘concentrations’ are typically assayed using the *Limulus* amoebocyte lysate assay (e.g. [1–3]). However, although satisfactory in simple matrices, this test is not considered very reliable in blood [4,5]. Indeed, because it is so hydrophobic, little or no LPS is actually free (unbound), and so it is not even obvious what its ‘concentration’ in blood might mean (see [5]). Although the quantitative assessment of LPS concentrations in WB can be problematic, its presence in this fluid may have important and clinically relevant effects on the blood microenvironment, and may be central in the treatment of inflammatory conditions [5–8].

LPS molecules are potent inflammagens (e.g. [9–11]) and may be both cytotoxic and/or neurotoxic [5,12–15]. They are known to induce the production of a variety of pro-inflammatory cytokines [16–19] that are involved in various apoptotic, programmed necrosis and pyroptotic pathways [5,20,21]. Indeed, cytokine production [16] is central to the development of inflammation [22]. A further characteristic of systemic inflammation is a hypercoagulatory state [23–27]. Such hypercoagulability is a common pathology underlying all thrombotic conditions, including ischaemic heart disease, ischaemic stroke and venous thromboembolism [28]. Furthermore, a hypercoagulable state is typically associated with pathological changes in the concentrations of fibrin(ogen) [29,30], and in particular an increase in the level of the fibrin degradation product D-dimer is seen as a reliable biomarker for cardiovascular risk [31,32].

Considering its cytokine-dependent effects, the question then arises as to whether LPS can cause hypercoagulation by acting on the coagulation pathway more directly. One route is via tissue factor (TF) upregulation; TF is related to the cytokine receptor class II family, and is *active early* in the (extrinsic) coagulation cascade, where it is necessary to initiate thrombin formation from prothrombin [33]. Recently, it was shown that LPS may upregulate TF; 100 ng ml^−1^ LPS added to healthy cord WB of newborns or the WB of healthy adults induced TF-mediated activation of haemostasis [34]. LPS from *Escherichia coli* (100 ng ml^−1^) also activated the coagulation system when added to WB, via a complement- and CD14-dependent upregulation of TF, leading to prothrombin activation and hypercoagulation [35]; however, this was noted after 2 h, and therefore it was not an acute process [35]. Note that in these studies, the anticoagulant was lepirudin, which prevents thrombin activation such that the effects of thrombin could not be evaluated. In this work, we used citrate as an anticoagulant.

**Figure 1.**
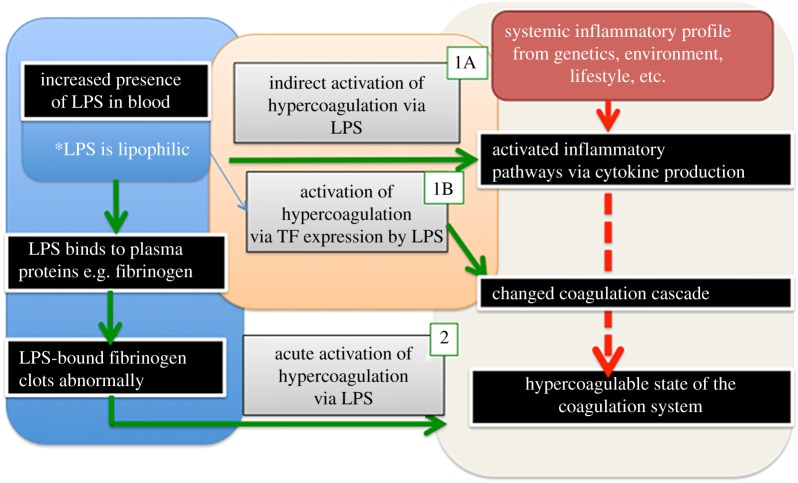
High-level effects of systemic inflammation on the coagulation system and the pathologic effects of LPS when present in blood and how it influences coagulation via a direct or indirect activation. Processes 1A and B are currently known for LPS activity in blood, while process 2 is a newly proposed acute reaction effect of LPS on blood and plasma. (Online version in colour.)

It occurred to us that, in addition to changes in TF expression by LPS, the process might also involve the direct binding of the lipophilic LPS to circulating plasma proteins, particularly fibrinogen, and that this (potentially rapid) binding might also cause pathological changes in the coagulation process. This would be independent of the slower TF activation, and thus an acute and relatively immediate process (figure 1). This indeed turned out to be the case. A preprint has been lodged at bioRxiv [36].

## 2. Results

### 2.1. Scanning electron microscopy of whole blood, plasma and purified fibrinogen

To investigate our hypothesis that LPS may cause hypercoagulation via an acute and direct binding reaction (by interaction with plasma proteins directly involved in the clotting cascade), we investigated the effect of two LPS preparations from *E. coli* (*viz*. O111:B4 and O26:B6). These were added to WB of healthy individuals, to platelet (and cell)-poor plasma (PPP), and to purified fibrinogen.

**Figure 2.**
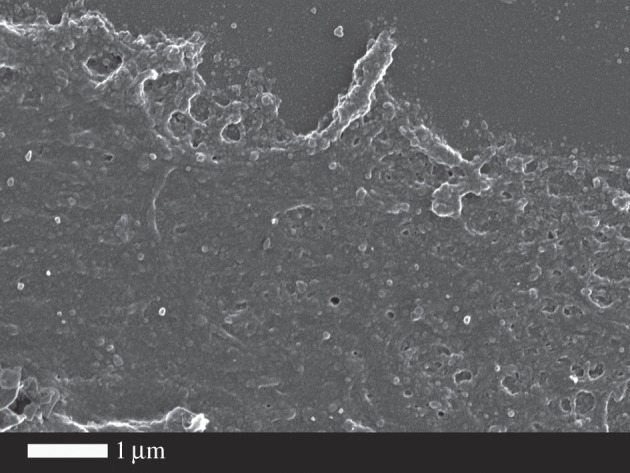
Effect of O111:B4 LPS (0.2 ng l^−1^) on whole blood (without thrombin), where dense matted deposits were spontaneously formed, not seen in control whole blood smears.

Although the physiological levels of LPS are said to be 10–15 ng l^−1^, and little or none of it is free [5], in our hands the addition of LPS at these concentrations caused immediate coagulation when they were added to WB. Figure 2 shows the effect of 0.2 ng l^−1^ O111:B4 LPS when added to WB and incubated for 10 min. Dense matted deposits are spontaneously formed; these are not seen in healthy WB. Fibrinogen with added O111:B4 or O26:B6 LPS with just 30 s exposure (no thrombin added) also spontaneously formed matted deposits (results not shown).

#### 2.1.1. Platelet poor plasma and lipopolysaccharide

**Figure 3.**
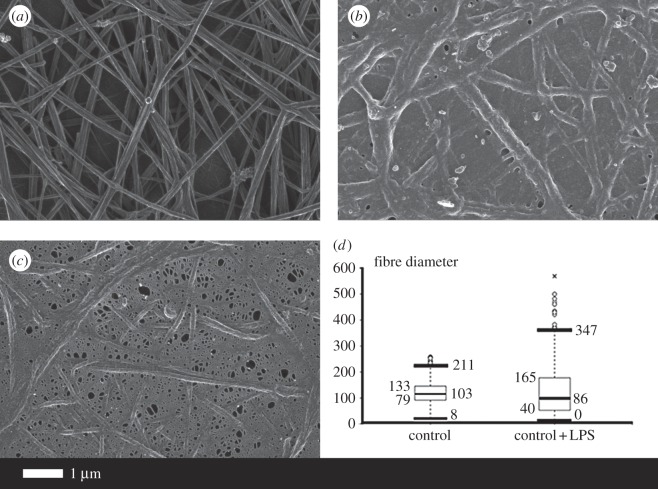
The effect of 0.2 ng l^−1^ O111:B4 LPS on the morphology of fibrin fibres in the platelet-poor plasma (PPP) of healthy individuals (with added thrombin). (*a*) Healthy fibres; (*b,c*) PPP with added LPS. (*d*) Fibre distribution of the control fibres and of controls with added LPS of 30 individuals. Note: in samples with added LPS, there were areas of matted layers with no visible fibres to measure. Fibres were measured using ImageJ as described in Material and methods.

Figure 3 shows the effects of thrombin on clot formation for a healthy control (figure 3*a*) and when the PPP was pre-incubated for 10 min with 0.2 ng l^−1^ O111:B4 LPS (two examples in figure 3*b,c*). Figure 3*d* shows the distribution of fibre thicknesses for the 30 individuals, with and without added LPS. The fibre thickness is much more heterogeneous after LPS is added. Clearly, these tiny amounts of LPS are having enormous effects on the clotting process. These kinds of netted structures, which we have also termed ‘dense matted deposits’, were previously seen in inflammatory conditions such as diabetes [37–39], iron overload and stroke [37,40–42]. Typically, healthy fibrin fibre networks form a net where individual fibrin fibres are seen, but with added LPS a matted net develops. There is a significant difference (*p* < 0.0001) between fibre thickness before and after LPS treatment in the presence of thrombin. Note, though, that the distribution of the fibre thickness in the LPS-treated group varies from very thick to very thin. In some cases, continuous fibre plates are formed, where no individual fibres could be seen or measured. This explains the difference in *n* between the ‘before’ and ‘after’ treatment (1450 versus 1330 measured fibres).

The experiment with the O111:B4 LPS was also repeated with a shorter 30 s exposure time. PPP with LPS and added thrombin showed fibre agglutination starting to happen in only 30 s of LPS exposure. These shorter experiments are to be contrasted with previously reported experiments that showed the much longer-term effect of LPS, involving cytokine production, including increased TF production via monocytes. By adding LPS to PPP (with thrombin), we bypass the possibility that LPS can stimulate TF production via the monocyte route suggested in [35,43]. To determine if another type of LPS would also cause the changes noted above, we also added *E. coli* O26:B6 LPS to PPP of five individuals (30 s and 10 min exposure time), followed by addition of thrombin. Scanning electron microscopy (SEM) results showed the same trends as noted with the O111:B4 LPS (results not shown).

#### 2.1.2. Confocal microscopy

**Figure 4.**
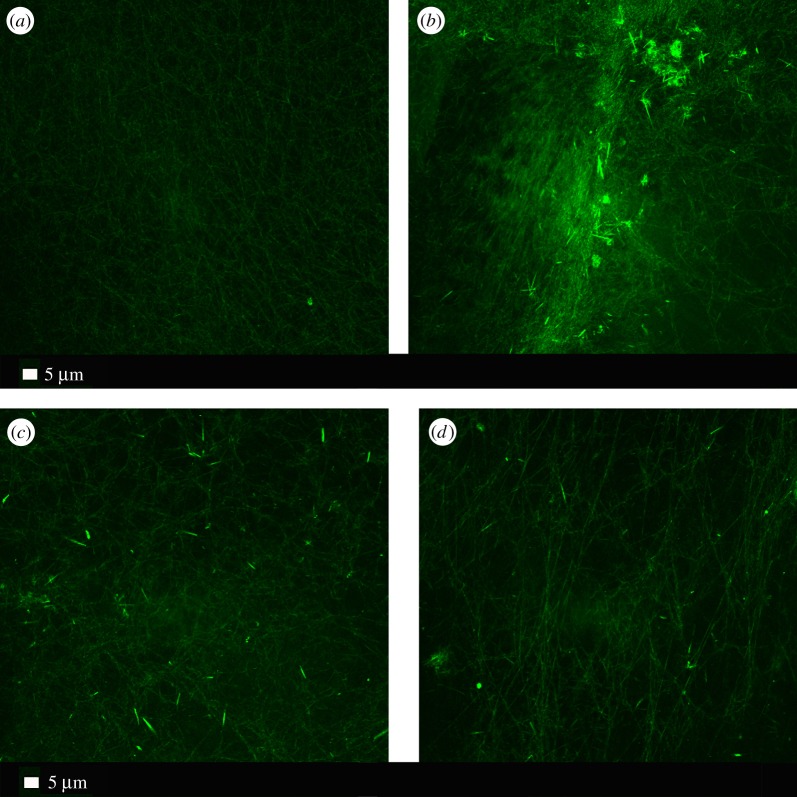
(*a*) Control PPP with ThT and thrombin and (*b*) as panel (*a*) but pre-incubated with 0.2 ng l^−1^ LPS; (*c*) as panel (*a*) but pre-incubated with 2 ng l^−1^ LBP; (*d*) as panel (*a*) but pre-incubated with 0.2 ng l^−1^ LPS and 2 ng l^−1^ LBP. (Online version in colour.)

The reaction, and presumed binding, of the hydrophobic LPS within fibrinogen fibres implies that they contain, or expose, significant hydrophobic elements. Such elements, also common in amyloid-like fibrils [44], can be stained fluorescently using dyes such as thioflavin T (ThT) [45]. We therefore studied the effect of 0.2 ng l^−1^ LPS on the ability of the fibrin fibres formed following thrombin treatment of PPP to bind ThT (figure 4). In contrast with the LPS-free controls (figure 4*a*), there is a very substantial binding of ThT to the fibrin fibres formed in the presence of the LPS (figure 4*b*). The lipopolysaccharide binding protein (LBP) is a potent binder of LPS, and would therefore be expected to inhibit the amyloidogenic effects on blood clotting observed. Thus, we also studied the effect of 2 ng l^−1^ LBP and 2 ng l^−1^ LBP + 0.2 ng l^−1^ LPS mixture on ThT binding (figure 4*c,d*) [7].

#### 2.1.3. Purified fibrinogen

**Figure 5.**
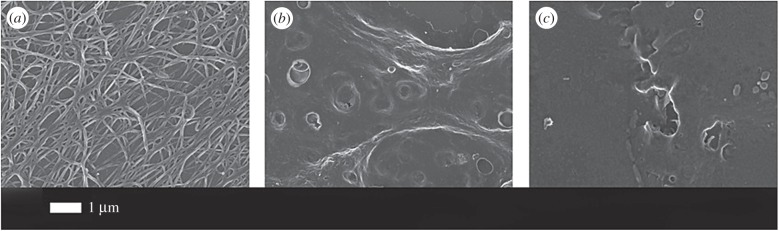
(*a*) Purified fibrinogen with added thrombin but no LPS; (*b*) purified fibrinogen with added O111:B4 LPS (30 s exposure) and 0.2 ng l^−1^ LPS; (*c*) as panel (*b*) but 10 min exposure.

It is worth rehearsing just how big an effect this is in molar terms: fibrinogen (MW 340 kDa) is present at in plasma at concentrations of approximately 2–4 g l^−1^ (Weisel's authoritative review [46] gives 2.5 g l^−1^), and its levels are increased during inflammation (see above), while the LPS (MW 10–20 kDa) was added at a concentration of 0.2 ng l^−1^. We will assume 15 kDa for the MW of LPS and 30 fg LPS per cell. Thus, 0.2 ng l^−1^ = 13 fM and 2.5 g l^−1^ fibrinogen approximately 7.35 µM which is a molar ratio of LPS; fibrinogen monomer in the WB of less than 10^−8^ : 1. As we are here only looking at the terminal stages of clotting, we considered that fibrinogen might be an important mediator of the LPS-induced hypercoagulation. Thus, we also added both LPS types to purified fibrinogen (30 s and 10 min exposure time) with added thrombin. Even the 30 s exposure time changed the fibrin fibres to form fibrils or dense matted deposits without any individual fibres visible (figure 5).

It is also worth rehearsing what 0.2 ng l^−1^ of LPS means in terms of the bacterial equivalents. Watson and co-workers [47] showed in laboratory cultures that LPS amounted to some 50 fg per cell in a logarithmic growth phase, falling to 29 fg per cell in stationary phase. As remarked previously [5], this shows at once that LPS contents per cell can be quite variable and that bacteria can shed a considerable amount of LPS at no major harm to themselves. On the basis that 1 mg dry weight of bacteria is about 10^9^ cells, each cell is about 1 pg, so 50 fg LPS per cell equates to about 5% of its dry weight, a reasonable and self-consistent figure for approximate calculations. We shall take the ‘starved’ value of 30 fg per cell. Thus, 0.2 ng l^−1^ (200 pg l^−1^) LPS is equivalent to the LPS content of approximately 7 × 10^3^ cells l^−1^. Most estimates of the dormant blood microbiome (that is derived mainly by dysbiosis from the gut and from the oral cavity as summarized in [5–7]) (some are much greater [48]) imply values of 10^3^–10^4^ ml, i.e. approximately 1000 times greater. In other words, a bacterial cell need lose only a small amount of its LPS to affect blood clotting in the way we describe here.

### 2.2. Isothermal titration calorimetry

**Figure 6.**
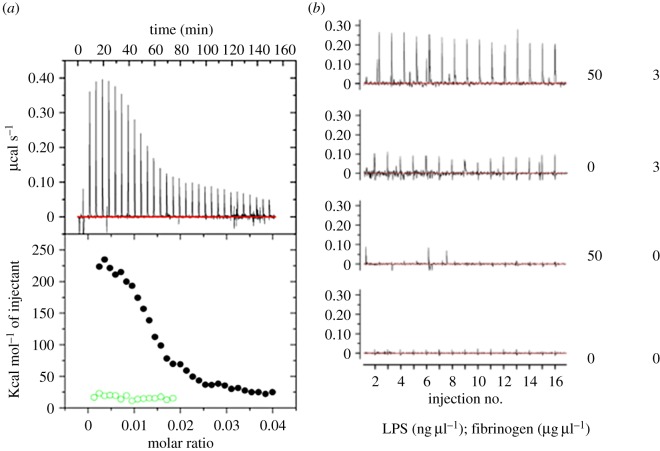
ITC analysis of the LPS–fibrinogen interaction. (*a*) Titration of 8.8 μM human plasma fibrinogen (*black circles*) or buffer (*green open circles*) into 100 µM of *E. coli* O111:B4 LPS. (*b*) Titration of 50 ng µl^−1^ LPS (2.5 µM) or buffer into 3 µg µl^−1^ fibrinogen (8.8 µM) or buffer as indicated. Experiments were conducted at 37°C in phosphate buffered saline. (Online version in colour.)

ITC is a sensitive and convenient method for detecting biomolecular interactions by measuring the heat that is released or absorbed upon binding [49]. Measurements are conducted directly in solution, without modification of immobilization of the interacting species. We used ITC to study potential interactions between human plasma fibrinogen and LPS from *E. coli* O111:B4. Titration of fibrinogen into LPS resulted in strong endothermic injection heats with a clear sigmoidal saturation curve indicating a direct binding interaction (figure 6*a*). Assuming molecular weights of 340 kDa for fibrinogen and 20 kDa for monomeric LPS, we determined a binding stoichiometry (*n*) of approximately 0.135. This is consistent with each fibrinogen monomer binding to a micelle formed from approximately 75 LPS monomers. Reverse titrations were conducted, injecting LPS into plasma concentrations of fibrinogen (3 g l^−1^ = 8.8 µM). Titration of 2.5 µM LPS into fibrinogen yielded endothermic injection heats greater than those observed for titration of 2.5 µM LPS into buffer alone (figure 6*b*), again clearly indicating a direct binding of LPS to fibrinogen. Each injection added 125 ng of LPS into the instrument cell, increasing the LPS concentration by approximately 30 nM per injection. Although we expect that LPS binds fibrinogen at sub-nanomolar concentrations, interactions at these concentrations are below the detection limits of the ITC instrument.

### 2.3. Thromboelastography of whole blood and platelet poor plasma

**Figure 7.**
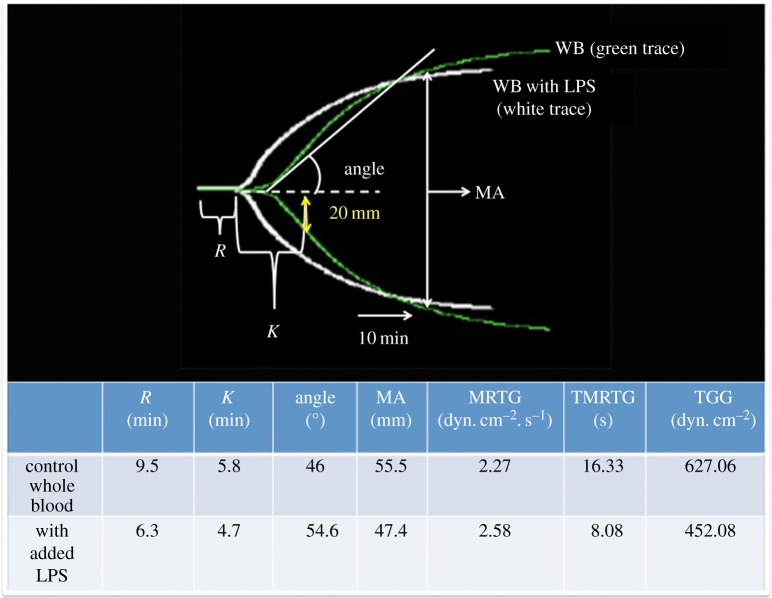
TEG overlay from a control whole blood sample with and without added LPS. *R*, reaction time, first measurable clot formation; *K*, achievement of clot firmness; angle, kinetics of clot development; MA, maximum clot strength; MRTG, maximum rate of thrombus generation; TMRTG, time to maximum rate of thrombus generation; TTG, final clot strength. (Online version in colour.)

Thromboelastography (TEG^®^) is a viscoelastic technique for measuring the clotting properties of WB [50,51]. TEG of WB and PPP was performed. TEG was not performed with purified fibrinogen because the coagulation activator in the TEG is CaCl_2_ and fibrinogen is only activated by thrombin, not calcium. Figure 7 shows a typical TEG trace from a control WB with and without added LPS, overlaid with lines that explain the parameters extracted by the instrument and the values for those traces. The statistics are given in table 1.

**Table 1.** Demographics of blood from healthy individuals with and without added LPS. Medians, standard deviation and *p*-values (values lower than 0.05 are indicated in italic) obtained using the Mann–Whitney *U*-test are shown for iron profiles, and TEG of whole blood and plasma. *R*, reaction time, first measurable clot formation; *K*, achievement of clot firmness; angle, kinetics of clot development; MA, maximum clot strength; MRTG, maximum rate of thrombus generation; TMRTG, time to maximum rate of thrombus generation; TTG, final clot strength.

variables	healthy individuals (*n*=30)	healthy individuals with added LPS (*n*=30)	*p*-value
age (years)	29.5 (±13.81)		
gender			
male	15 (50%)		
female	15 (50%)		
iron profiles			
iron µM	16.9 (±6.18)		
transferrin g l^−1^	2.75 (±0.46)		
% saturation	25.0 (±10.94)		
serum ferritin ng ml^−1^	42.5 (±92.47)		
fibrin fibre thickness	*n* = 1450	*n* = 1330	
fibre thickness (nm)	103 (±40)	86 (±82)	*<0.0001*
TEG^®^			
TEG of whole blood—recalcified with CaCl_2_ with added O111:B4 LPS (10 min)			
MRTG (dyn cm^−2^ s^−1^)	2.61 (±1.13)	2.89 (±0.90)	0.33
TMRTG (min)	13.9 (±3.53)	9.6 (±3.01)	*<0.0001*
TTG (dyn cm^−2^)	615.0 (±179.55)	527.9 (±146.65)	*0.049*
*R* (min)	8.0 (±1.64)	6.2 (±1.77)	*<0.0001*
*K* (min)	4.9 (±2.63)	4.2 (±1.23)	0.07
angle (°)	49.8 (±5.27)	56.2 (±7.02)	*0.0066*
MA (mm)	55.0 (±8.07)	51.3 (±6.90)	0.092
TEG of platelet-poor plasma—recalcified with CaCl_2_ with added O111:B4 LPS (10 min)			
MRTG (dyn cm^−2^ s^−1^)	3.6 (±4.35)	4.2 (±2.13)	0.36
TMRTG (min)	10.6 (±3.22)	9.3 (±3.68)	0.50
TTG (dyn cm^−2^)	203.9 (±137.51)	211.6 (±103.67)	0.70
*R* (min)	8.2 (±2.64)	7.1 (±2.70)	*0.026*
*K* (min)	4.4 (±3.51)	3.8 (±2.42)	0.18
angle (°)	63.2 (±2.70)	54.4 (±10.67)	0.23
MA (mm)	28.4 (±8.34)	30.1 (±9.27)	0.196
TEG of platelet-poor plasma—recalcified with CaCl_2_ with added O111:B4 LPS (30 s)			
	*n* = 5	*n* = 5	
MRTG (dyn cm^−2^ s^−1^)	5.94 (±1.8)	8.2 (±2)	0.166
TMRTG (min)	11.58 (±1.2)	9 (±1.3)	*0.0159*
TTG (dyn cm^−2^)	244.4 (±69.9)	290.2 (±66.5)	>0.99
*R* (min)	9.8 (±1.2)	7 (±1.5)	*0.031*
*K* (min)	2.8 (±1.6)	2 (±0.9)	0.119
angle (°)	63.6 (±6.5)	68.8 (±8.2)	0.095
MA (mm)	32.8 (±6.5)	36.7 (±5.5)	>0.99
TEG of platelet-poor plasma—recalcified with CaCl_2_ with added O26:B6 LPS (30 s)			
	*n* = 5	*n* = 5	
MRTG (dyn cm^−2^ s^−1^)	6.3 (±2.6)	6.2 (±3.6)	0.70
TMRTG (min)	11.6 (±2.1)	8.9 (±1.5)	0.15
TTG (dyn cm^−2^)	276.7 (±43.1)	230.8 (±202.2)	0.69
*R* (min)	9.8 (±1.5)	6.4 (±1.4)	*0.05*
*K* (min)	2.1 (±0.5)	2.1 (±1.1)	0.88
angle (°)	64.4 (±4.3)	70.8 (±10.4)	>0.99
MA (mm)	35.5 (±3.3)	31.5 (±13)	0.60

#### 2.3.1. Whole blood

TEG analysis of WB (10 min incubation time with O111:B4 LPS) showed that the *R*, TMRTG and TTG are all significantly decreased (table 1). Changes to *R* indicate that the clot forms quicker and a decreased TMRTG indicates that the time to maximum thrombus generation is also faster, suggesting a hypercoagulable state. The TTG is also significantly decreased suggesting total thrombus generation, thus implying clot strength is decreased, although the clot forms faster. The SEM fibrin fibre thickness results show areas where no individual fibres are formed; instead, a matted homogeneous layer forms, and there are also areas of fine and short fibres. Here, we suggest that this morphology is probably related to the decreased TTG, where the fibrin structure results in a clot with decreased strength. We have not measured lyses, but a decreased TTG is likely to indicate a hypofibrinolytic nature of the clot. We have previously shown that the same concentration of LPS as used in this paper, when added to naive uncitrated healthy blood but *without added CaCl_2_*, also had an effect on coagulation after only 30 s incubation time [52]. Both TMRTG and *R* of naive WB were also significantly shorter, also showing hypercoagulation, and the TTG was increased, but not significantly increased. However, in this study, the blood was not drawn in citrated tubes and therefore not re-calcified.

#### 2.3.2. Platelet-poor plasma

We also performed similar TEG experiments with PPP (table 1). After 10 min exposure, just the initial clotting time *R* was changed. The results were not specific for O111:B4 LPS as O26:B6 LPS added to PPP behaved similarly. The decreased *R* time is indicative of reaction time, and therefore the time to first measurable clot formation is also significantly decreased (as the initiation of the clot starts faster with than without LPS), confirming a hypercoagulable state with added LPS. After 30 s exposure time, both the *R* and the TMRTG were shorter (in the five patients tested). This confirms our hypothesis that LPS causes (near) instant hypercoagulability. Here also, the results were not specific for O111:B4 LPS as O26:B6 LPS added to PPP also showed a decreased *R*-time. TTG of PPP was increased (but not significantly increased) after 30 s, as well as 10 min, which compared with results of PPP from patients with Alzheimer's type dementia [52].

Table 1 shows a summary of the various viscoelastic properties (TEG experiments) after LPS has been added to WB and PPP. There are very substantial changes in a number of the clotting parameters. Following these 10 min exposure experiments, we shortened our experimental time to 30 s and we repeated the experiments with five samples, using PPP, where significant changes were still observed. The results were not specific for O111:B4 LPS as O26:B6 LPS. We note that WB with added LPS showed a more pronounced change in relevant viscoelastic parameters than when LPS was added to PPP (table 1).

## 3. Discussion

In the introduction, we suggested that LPS might contribute to excessive blood clotting (or an activated coagulation state) via two possible routes: (i) via a direct and acute binding to plasma proteins (e.g. fibrinogen) or (ii) by an indirect or chronic (longer-term) process where it participates in an inflammatory activation via cytokine production. Here, we showed that the first process is indeed possible, using tiny amounts of LPS that amounted in molar terms to less than 10^−8^ relative to fibrinogen, and demonstrated it by both viscoelastic and ultrastructural methods. We also confirmed that LPS can change the viscoelastic properties of PPP within 30 s of its addition. Furthermore, WB with added LPS, but without thrombin activation, showed spontaneously formed, amyloid-like matted deposits. Purified fibrinogen experiments with O111:B4 LPS and O26:B6 LPS, with and without added thrombin showed a changed ultrastructure, suggesting that LPS indeed binds to the 340 kDa fibrinogen molecule and that the effects of this are visible ultrastructurally.

LPS, and especially its lipid A component, is highly lipophilic, and it therefore may be able to bind directly to plasma proteins, in an acute way. This might be one reason underlying the hypercoagulability [5], as well as a denser clot structure [53,54], as seen in various inflammatory diseases. Although we show here that exposure to even tiny amounts of LPS leads to an immediate (acute) change in the coagulability parameters, we recognize that this may happen simultaneously with chronic (longer-term) reactions (figure 1). Fibrinogen molecules are roughly 5×45 nm, and their self-assembly is a remarkable process (some 5800 are involved in generating a fibre of 80–90 nm diameter and 1 µm length). This would explain why the highly substoichiometric binding of LPS can have such considerable effects, especially as observed in WB. Following Anfinsen [55], it is assumed that most proteins adopt their conformation of lowest free energy. However, this is not true for amyloid fibre formation [56] nor in the case of the autocatalytic conversion of prion protein conformations [57,58]. At present, the exact mechanisms of action of these small amounts of LPS are not known, although it is indeed simplest to recognize fibrinogen polymerization as a cascade effect, much as occurs for amyloid and prion proteins whose initial conformation is not in fact that of their lowest free energy [59]. Specifically [60], the ‘normal’ conformational macrostate of such proteins is not in fact that of the lowest free energy, and its transition to the energetically more favourable ‘rogue’ state is thermodynamically favourable but under kinetic control, normally (in terms of transition state theory) with a very high energy barrier Δ*G*^†^ of maybe 36–38 kcal mol^−1^ [60]. Indeed, it is now known that quite a number of proteins of a given sequence can exist in at least two highly distinct conformations [61]. Typically the normal (‘benign’) form, as produced initially within the cell, will have a significant α-helical content, but the abnormal (‘rogue’) form, often in the form of an insoluble amyloid, will have a massively increased amount of β-sheet [62], whether parallel or antiparallel. In the case of blood clotting, we at least know that this is initiated by the thrombin-catalysed loss of fibrinopeptides from fibrinogen monomers (e.g. [41,63]). The massive adoption of a β-sheet conformation, as revealed here for the first time by the thioflavin T staining, demonstrates directly that virtually every fibrinogen molecule in the fibrin fibril must have changed its conformation hugely; it is not just a question of static ‘knobs and holes’ as usually depicted. We also showed that LBP, and a mixture of LPS and LBP, shows decreased ThT binding, compared with LPS alone.

Previously, we coined the term ‘atopobiotic’ microbes to describe microbes that appear in places other than where they should be, e.g. in the blood, forming a blood microbiome [6]. Here, we suggest that the metabolic and cell membrane products of these atopobiotic microbes correlate with, and may contribute to, the dynamics of a variety of inflammatory diseases [64–67], and that LPS, in addition to (possibly low-grade) long-term inflammation via cytokine production, may lead an acute and direct hypercoagulatory effect by binding to plasma proteins, especially fibrinogen. Specifically, we showed here that, even with very low levels and highly substoichiometric amounts of LPS, a greatly changed fibrin fibre structure is observed. An urgent task now is to uncover the mechanism(s) of this acute and immediate effect, with its remarkable molecular amplification.

## 4. Material and methods

### 4.1. Sample population

In total, 30 healthy individuals were included in the study. Exclusion criteria were known inflammatory conditions such as asthma, human immunodeficiency virus (HIV) or tuberculosis, and risk factors associated with metabolic syndrome, smoking, and, if female, being on contraceptive or hormone replacement treatment. Full iron tests were performed, as high serum ferritin and low transferrin levels are acute phase inflammatory protein markers [68] and indicative of inflammation. We included controls only if their iron levels were within normal ranges. WB of the participants was obtained in citrate tubes and either WB or platelet-poor plasma was used in this study for TEG, confocal and SEM experiments.

### 4.2. Lipopolysaccharide types, purified fibrinogen and thrombin concentration used

The LPS used was from *E. coli* O111:B4 (Sigma, L2630) and also *E. coli* O26:B6 (Sigma L2762). A final LPS exposure concentration of 0.2 ng l^−1^ (well below its critical micelle concentration [69]) was used in all experiments bar as noted for some of the ITC measurements. A final LBP exposure concentration of 2 ng l^−1^ LBP and a mixture with final exposure concentration of LPS (0.2 ng l^−1^) and LBP (2 ng l^−1^), incubated for 10 min with PPP, were also used (only confocal studies).

For ITC experiments, a micellar suspension of 10 mg l^−1^ was vortexed, followed by multiple serial dilutions. The South African National Blood Service (SANBS) supplied human thrombin, which was at a stock concentration of 20 U ml^−1^ and was made up in a PBS containing 0.2% human serum albumin. In experiments with added thrombin, 5 µl of thrombin was added to 10 µl of PPP or fibrinogen (with and without LPS exposure). Human fibrinogen was purchased from Sigma (F3879–250MG). A working solution of 0.166 mg ml^−1^ purified fibrinogen was prepared. This concentration was found to be the optimal concentration to form fibrin fibres in the presence of thrombin, similar to that of platelet-rich plasma fibres from healthy individuals [70]. As noted by a referee, LPS is a common laboratory contaminant, and care is needed; however, this was not an issue here as the ‘no-added-LPS’ controls showed.

### 4.3. Addition of lipopolysaccharide ± thrombin to whole blood, plasma and purified fibrinogen

LPS-incubated WB and purified fibrinogen were prepared for SEM without added thrombin (LPS exposure concentration: 0.2 ng l^−1^). LPS-incubated PPP and purified fibrinogen samples were prepared as above, but with added thrombin to create an extensive fibrin fibre network (also with LPS exposure concentration: 0.2 ng l^−1^ before addition of thrombin).

### 4.4. Isothermal titration calorimetry

*E. coli* O111:B4 LPS and human plasma fibrinogen were purchased from Sigma-Aldrich. Samples were reconstituted in warm phosphate buffered saline and incubated for 1 h at 37°C with shaking. LPS was then sonicated for 1 h at 60°C. Fibrinogen solutions were passed through a 0.2 µm polyethersulfone syringe filter and concentrations were determined by UV absorbance (*E*_1%_ = 15.1 at 280 nm). Samples were then diluted with buffer to the required concentration and degassed. ITC experiments were performed at 37°C on a MicroCal Auto-iTC200 system (GE Healthcare) in high-gain mode at a reference power of 10 µcal s^−1^, with an initial 0.5 µl (1 s) injection followed by 15 2.5 µl (5 s) injections with 300 s spacing. For longer titrations, the syringe was refilled and injections continued into the same cell sample. Control runs were performed in which cell samples were titrated with buffer and syringe samples were titrated into buffer, and data from these runs were subtracted from the experimental data as appropriate. Data analysis was performed in Origin, using the supplied software (MicroCal).

### 4.5. Thromboelastography

TEG was used to study the viscoelastic properties of the participants' blood, before and after addition of LPS. WB TEG was performed on day of collection (after 10 min incubation time with LPS—final exposure concentration 0.2 ng l^−1^ and PPP was stored in 500 µl aliquots in a −70°C freezer. The thawed citrated PPP (with and without LPS—where LPS was added to PPP at a final exposure concentration of 0.2 ng l^−1^. The incubation time of LPS and PPP was 10 min, as with WB. Standard TEG procedures were followed with addition of CaCl_2_ to activate the coagulation process as previously described [50,51,71,72]. TEG was also performed on 5 PPP samples, 30 s after adding O111:B4 LPS or O26:B6 LPS.

### 4.6. Confocal microscopy

Thioflavin T (ThT) was added at an exposure concentration of 5 µM to 200 µl of PPP (incubated for 1 min, and protected from light). A second sample was also prepared by adding an exposure concentration of 0.2 ng l^−1^ LPS (incubate for 10 min, at room temperature) before the addition of ThT. After an incubation time of 1 min and incubation protected from light, 10 µl of the PPP (with and without LPS) was mixed with 5 µl of thrombin (see SEM preparation of extensive fibrin fibres—as described above). To determine if ThT binding will happen in the presence of LBP, 2 ng l^−1^ LBP was pre-incubated for 10 min with PPP, followed by 1 min ThT exposure, and fibrin fibre preparation by adding thrombin. A mixture of LPS and LBP was also made (final PPP exposure concentration: 0.2 ng l^−1^ LPS and 2 ng l^−1^ LBP). PPP was exposed for 10 min to this mixture, followed by a 1 min exposure of ThT and fibrin fibre formation by adding thrombin to mixture-exposed PPP. Samples were viewed under a Zeiss LSM 510 META confocal microscope with a Plan-Apochromat 63×/1.4 Oil DIC objective, excitation was at 488 nm and emission measured at 505–550.

### 4.7. Statistical analysis

The non-parametric Mann–Whitney *U*-test was performed using STATSDIRECT software.
